# Identification of Clade E Avipoxvirus, Mozambique, 2016

**DOI:** 10.3201/eid2309.161981

**Published:** 2017-09

**Authors:** Lourenço P. Mapaco, Zeiss Lacerda, Iolanda V.A. Monjane, Esayas Gelaye, Afonso H. Sussuro, Gerrit J. Viljoen, William G. Dundon, Sara J. Achá

**Affiliations:** Agrarian Research Institute of Mozambique, Maputo, Mozambique (L.P. Mapaco, I.V.A. Monjane, A.H. Sussuro, S.J. Achá);; Eduardo Mondlane University, Maputo (Z. Lacerda);; National Veterinary Institute, Debre Zeit, Ethiopia (E. Gelaye);; International Atomic Energy Agency, Vienna, Austria (G.J. Viljoen, W.G. Dundon)

**Keywords:** avipoxvirus, clade E, fowlpox, phylogenetic analysis, Mozambique, Africa, poultry, chicken, turkey, vaccine, viruses

## Abstract

Analysis of scab samples collected from poultry during outbreaks of fowlpox in Mozambique in 2016 revealed the presence of clade E avipoxviruses. Infected poultry were from flocks that had been vaccinated against fowlpox virus. These findings require urgent reevaluation of the vaccine formula and control strategies in this country.

Avipoxviruses are large, enveloped DNA viruses that belong to the genus *Avipoxvirus* in the *Chordopoxvirinae* subfamily of the family *Poxviridae*. These viruses cause disease in a large number of bird species and are generally named after the species from which the virus was first isolated and characterized (*1*). Fowlpox virus (FPV) has caused substantial economic losses in domestic poultry resulting from reduced egg production and growth, blindness, and death, with a death ratio that can reach as high as 50%.

Phylogenetic analyses of the *Avipoxvirus* genus are usually conducted with the segments of the genes encoding the 4b core-like protein (P4b) and the DNA polymerase, which are both highly conserved among poxviruses (*2**,**3*). Using these loci, researchers have seen that most strains cluster into 3 major clades, namely A, B, and C, with clade A being subdivided further into subclades A1–A7 and clade B into subclades B1–B3 (*3**–**5*). Two additional clades (i.e., D and E), each with just a single isolate, have also been proposed (*5**,**6*).

Little is known about avipoxviruses in Africa. Avipoxviruses isolated from chickens, turkeys, and pigeons in 2011 in northern Egypt belonged to either subclade A1 or A2 (*7*). In 2013, thirteen avipoxviruses from different bird species from different regions of South Africa grouped into either subclade A2 or A3 (*8*). Sequences generated from isolates from naturally infected chickens in Tanzania were also found to belong to clade A1 (*9*). Therefore, all avipoxviruses previously identified in Africa belonged to clade A.

Fowlpox is endemic in Mozambique and commonly reported. The effect of the disease is more severe in backyard production systems affecting mostly young chickens and turkeys. An official control program for FPV in Mozambique does not exist, and because the country does not have a poultry production system that meets the national demand, birds are often imported from neighboring countries, such as South Africa, Swaziland, and Zimbabwe.

During August 2015–November 2016, scab samples from 16 separate FPV outbreaks were collected by the Agrarian Research Institute of Mozambique from 4 locations: Gaza Province, Maputo Province, Maputo City, and Tete Province. The outbreaks primarily affected backyard chickens and commercial laying hens, although a flock of broiler chickens and a flock of turkeys were also investigated. The clinical signs reported and postmortem examination findings included reduction of appetite; listlessness; nodules or scabs of different sizes on less-feathered body areas (e.g., wattles, comb, eye lids, ear lobes, limbs, and interdigit spaces); and pronounced ulcerations on the interdigit space. Different color tones and irregular wrinkled shells were also observed on eggs.

The 16 scab samples were positive for FPV P4b gene following PCR amplification with specific primers (Technical Appendix); the amplicons were purified and sequenced. A phylogenetic analysis of the P4b gene sequences revealed that most of the samples collected contained virus that clustered in subclade A1 (Figure, panel A). However, the 2 samples that did not cluster in subclade A1 (FPV-MOZ-608/2016 and FPV-MOZ-980/2016) were of interest; these samples were obtained from 2 separate outbreaks in layer chickens 3 months apart (i.e., in Maputo City [25°55′55.5′′S, 32°32′53.4′′E] on July 7, 2016, and in Maputo Province [25°53′22.3′′S, 32°27′16.6′′E] on October 25, 2016). To characterize FPV-MOZ-608/2016 and FPV-MOZ-980/2016 further, we amplified and sequenced a segment of the DNA polymerase gene (Technical Appendix). The maximum-likelihood analysis using both P4b and DNA polymerase gene fragments showed that FPV-MOZ-608/2016 and FPV-MOZ-980/2016 clustered in clade E with the APV isolated in Hungary in 2011 (TKPV-HU1124/2011) (Figure, panels A and B) (*6*).

**Figure:**
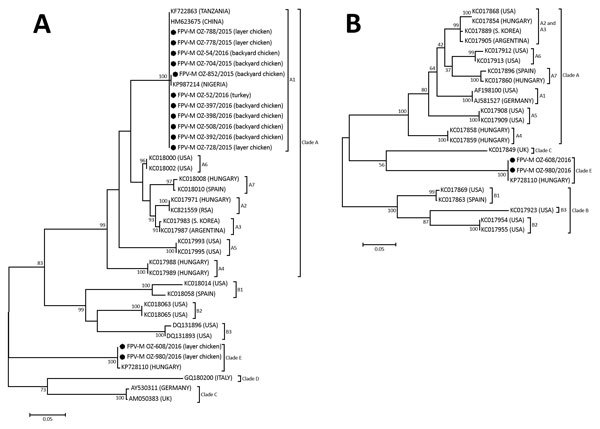
Phylogenetic analyses of avipoxviruses from 16 outbreaks, Mozambique, August 2015–November 2016. A) 4b core-like protein gene fragment. B) DNA polymerase gene fragment. Black circles indicate samples sequenced in this study. GenBank accession numbers and country of origin are indicated for related sequences. Evolutionary analyses were conducted with MEGA6 (http://www.megasoftware.net). The bootstrap values from 500 replicates are shown at nodes. Scale bars indicate number of nucleotide substitutions per site.

TKPV-HU1124/2011 was isolated from a flock of turkeys vaccinated with a commercial vaccine for FPV, and FPV-MOZ-608/2016 and FPV-MOZ-980/2016 were also obtained from vaccinated chickens. The laying pullets imported from South Africa had already been vaccinated for FPV on day 1 with the commercial fowlpox-vectored infectious laryngotracheitis vaccine and day 17 with the fowlpox-vectored infectious laryngotracheitis/avian encephalomyelitis vaccine. Our data suggest a possible failure of the vaccine to protect against clade E viruses, similar to what has been reported previously for TKPV-HU1124/2011 (*6*).

The identification of a clade E avipoxvirus in Mozambique requires further investigation to clarify how a virus that has only been reported once found its way to this country. Because the chickens in both infected flocks were purchased from the same pullet reseller who had (for both flocks) imported the birds from South Africa, it is likely that the source of infection was the same. However, the specific source has not been identified. FPVs are known to infect >230 species of wild birds, many of which are migratory (*5*); thus, introduction through migratory wild birds is a possibility.

Resolution of the full genome of these viruses might provide hints to their origin. The presence of fowlpox disease in birds vaccinated against FPV requires urgent reevaluation of the vaccine formula and control strategies in Mozambique.

Technical Appendix Description of the methods used for DNA isolation and PCR amplification and sequencing of the 4b core-like protein and DNA polymerase gene fragments of avipoxviruses, Mozambique.
