# Supplementary material for: Identification of Clade E Avipoxvirus, Mozambique, 2016
Source: Emerg Infect Dis. 2017 Sep;23(9):1602–4. doi: 10.3201/eid2309.161981 (PMC5572868; doi:10.3201/eid2309.161981)
Supplement: Technical Appendix — Description of the methods used for DNA isolation and PCR amplification and sequencing of the 4b core-like protein and DNA polymerase gene fragments of avipoxviruses, Mozambique. [file 16-1981-Techapp-s1.pdf]

Article DOI: https://doi.org/10.3201/eid2309.161981

Identification of Clade E Avipoxvirus, Mozambique, 2016

Technical Appendix

DNA Isolation, Amplification, and Sequencing

DNA was extracted directly from scab samples collected from different parts of birds (e.g., wattles, comb, eye lids, ear lobes, limbs, and interdigital spaces) by using a DNeasy Blood & Tissue Kit (QIAGEN, Hilden, Germany). A 368-bp fragment of the 4b core-like protein gene was amplified by PCR with the primer pair FP-For (5′-CAGCAGGTGCTAAACAACAA-3′) and FP-Rev (5′-CGGTAGCTTAACGCCGAATA-3′) (*1*). A 1,000-bp fragment of the DNA polymerase was amplified by PCR with the primer pair PPolF (5′-GGCYAGTACKCTTATYAAAGG-3′) and PPolR (5′-CGTCTCTACGTGTTTCGCT-3′) (*2*).

The following thermal profile was used for both PCR amplifications: initial denaturation at 95°C for 5 min; 35 cycles of denaturation at 95°C for 30 s, annealing at 55°C for 35 s, and elongation at 72°C for 45 s; followed by a final elongation at 72°C for 7 min. Positive PCR amplicons were purified with a Wizard SV Gel and PCR Clean-Up System (Promega, Madison, WI, USA) and were sent to LGC Genomics (Berlin, Germany) for sequencing with the same primers used for the amplification. All 4b core-like protein and DNA polymerase gene sequences generated here were deposited in GenBank (accession nos. KX988302 and KY312501–KY312503).

Phylogenetic Analysis

The Staden Package (http://staden.sourceforge.net/) was used to assemble the generated sequences. Multiple sequence alignment was performed by using MUSCLE (http://www.ebi.ac.uk/Tools/msa/muscle/) with default settings, incorporating all the sequences generated here combined with those available in GenBank. This resulted in a final data set of 41 sequences 368 bp in length for the 4b core-like protein gene fragment and 23 sequences 542 bp

in length for the DNA polymerase gene. A phylogenetic tree was estimated by using the maximum likelihood method available in MEGA6 (*3*), the Kimura 2-parameter model of nucleotide substitution, and 500 bootstrap replications.

References

1. Jarmin S, Manvell R, Gough RE, Laidlaw SM, Skinner MA. Avipoxvirus phylogenetics: identification of a PCR length polymorphism that discriminates between the two major clades. J Gen Virol. 2006;87:2191–201. PubMed http://dx.doi.org/10.1099/vir.0.81738-0

2. Gyuranecz M, Foster JT, Dán Á, Ip HS, Egstad KF, Parker PG, et al. Worldwide phylogenetic relationship of avian poxviruses. J Virol. 2013;87:4938–51. PubMed http://dx.doi.org/10.1128/JVI.03183-12

3. Tamura K, Stecher G, Peterson D, Filipski A, Kumar S. MEGA6: Molecular Evolutionary Genetics Analysis version 6.0. Mol Biol Evol. 2013;30:2725–9. PubMed http://dx.doi.org/10.1093/molbev/mst197
